# Differential Expression and Functional Analysis of CircRNA in the Ovaries of Low and High Fecundity Hanper Sheep

**DOI:** 10.3390/ani11071863

**Published:** 2021-06-23

**Authors:** Aiju Liu, Xiaoyong Chen, Menghe Liu, Limeng Zhang, Xiaofei Ma, Shujun Tian

**Affiliations:** 1 Department of Animal Genetics, Breeding and Reproduction, College of Animal Science and Technology, Hebei Agricultural University, Baoding 071000, China; liuaiju890526@163.com (A.L.); chenxiaoyong-2000@163.com (X.C.); ma19951116@126.com (X.M.); 2 Discipline of Obstetrics and Gynaecology, School of Medicine, Robinson Research Institute, University of Adelaide, Adelaide, SA 5005, Australia; menghe.liu@adelaide.edu.au; 3 Laboratory of Molecular Biology, Zhengzhou Normal University, Zhengzhou 450000, China; zlmklmyt@163.com; 4 The Research Center of Cattle and Sheep Embryonic Technique of Hebei Province, Baoding 071000, China

**Keywords:** fecundity, functional enrichment, Hanper sheep, circRNAs, ovary

## Abstract

**Simple Summary:**

Litter size is an important trait affecting reproductive capacity and breeding economics in meat sheep. Consequently, revealing its molecular mechanism helps us understand multiple lambs from the genetic perspective. In this study, we provide a genome-wide expression profile of circular RNAs (circRNAs) expression in Hanper sheep, which is a new breed of meat sheep raised by cross and self-group breeding for 15 years. In this study, ovarian circular RNAs and miRNAs associated with high and low fertility Hanper sheep are identified during the follicular and luteal phases of the estrous cycle, and their potential biological functions are predicted through Gene Ontology (GO), KEGG, GSEA, STEM, WGCNA analysis.

**Abstract:**

Litter size is a considerable quality that determines the production efficiency of mutton sheep. Therefore, revealing the molecular regulation of high and low fertility may aid the breeding process to develop new varieties of mutton sheep. CircRNAs are the important factors regulating follicular development, but their mechanism role in the regulation of litter size in Hanper sheep is not clear. In the present study, ovarian tissues from the follicular (F) or luteal phase (L) of Hanper sheep that were either consecutive monotocous (M) or polytocous were collected. Then, we performed transcriptome sequencing to screen for differentially expressed circRNAs (DE-circRNAs) and elucidate their function. In total, 4256 circRNA derived from 2184 host genes were identified in which 183 (146 were upregulated, while 37 were downregulated) were differentially expressed in monotocous sheep in the follicular phase versus polytocous sheep in the follicular phase (MF vs. PF). Moreover, 34 circRNAs (14 were upregulated, while 20 were downregulated) were differentially expressed in monotocous sheep in the luteal phase versus polytocous sheep in the luteal sheep (ML vs. PL). This was achieved through DE-circRNAs function enrichment annotation analysis by GESA, GO, and KEGG, which function through the EGF-EGFR-RAS-JNK, TGF-β and thyroid hormone signaling pathway to affect the litter size of Hanper sheep in MF vs. PF and ML vs. PL. STEM results showed that MAPK signaling pathways play a key role in MF vs. PF and ML vs. PL. Through WGCNA analysis, *AKT3* was a core gene in MF vs. PF and ML vs. PL. Moreover, competitive endogenous RNA (ceRNA) network analysis revealed the target binding sites for miRNA such as oar-miR-27a, oar-miR-16b, oar-miR-200a/b/c, oar-miR-181a, oar-miR-10a/b, and oar-miR-432 in the identified DE-cirRNAs.

## 1. Introduction

Litter size is an important trait affecting reproductive capacity and breeding economics in meat sheep. Consequently, revealing its molecular mechanism helps us understand multiple lambs from the genetic perspective. For example, FecB mutation, which is closely related to litter size, has a significant effect on small metabolic molecules in follicular fluid, making the follicular fluid stronger in its own antioxidant defense. From genomic, transcriptome, and proteomic levels, we found that FecB mutation promoted the expression time of the *LHCGR* gene on cumulus granulosa cells affecting the proliferation of cumulus granulosa cells [1]. Through miRNA and lncRNA analysis of FecB ^BB^ Small Tail Han sheep and FecB++ Dorset sheep, it was found the differentially expressed genes on cell apoptosis and proliferation were affected by gene mutation [2,3] and participated in progesterone mediated steroid biosynthesis and oocyte meiosis pathway [4]. *Xist* (loc101112291) and *Gtl2* (loc101123329) were found to be highly expressed in Hanper sheep, suggesting the regulation of follicular development through methylation processes [5]. Mir-6404 and mir-29c are involved in the regulation of high and low fecundity in goats [6]. The expression of *CTSB* and *CSSD* in the ovarian tissue of Hu sheep with high and low fertility at the follicular stage was significantly up-regulated (*p* < 0.05) [7]. Comparative proteomics showed that taurine, sulfite metabolism, and apoptosis were involved in the regulation of ovarian function and closely related to follicular development and ovulation, revealing that FecB mutation was related to the high expression of mitochondrial oxidation related proteins [8]. In addition, the weighted gene co-expression network analysis showed that *NR0B1*, XLOC_041882, and *MYH15* were mainly involved in the TGF-β signaling pathway, while *NYAP1* and *BCORL1* were significantly correlated with the oxytocin signaling pathway [9].

Ovary, the place where oocytes are produced and reproductive hormones are secreted, is directly related to follicular development and ovulation number. Previously, ncRNAs were regarded as a type of junk RNA that does not get translated. With the advancement of ultra-deep sequencing technology and bioinformatics, the functions of ncRNA became increasingly evident involving epigenetic and transcriptional regulations. Especially, the ncRNA mediated transcriptional regulation has gained tremendous attention. Different kinds of ncRNAs, namely the miRNA [10], LincRNA [11], and circRNA have been identified [12]. Among these, circRNAs are known to regulate various biological processes and organ function [13], such as pituitary [14], hypothalamus [15], muscle [16,17], spleen [18], and so on. However, the function of circRNAs in Hanper sheep ovaries is still unclear, in particular, their role in regulating the litter size is largely unknown. CircRNAs are mainly formed by pre-mRNA reverse splicing [19] and are composed of an intron sequence [20], RNA binding protein [21], typical RNA splicing signal [22], and lasso precursor containing exons [23]. CircRNAs perform various biological functions, including acting as sponges for miRNAs [12], for example, testis-specific sex determination region Y (*SRY*) 9 sponges miR-138 [24]. *CircDDXlO* sponges miR-1301-3p or miR-4660 to control sirtuin 3 (*SIRT3*) expression, regulating the aging of human ovaries [25]. In bovine cumulus cells, circRNA_11396 functions as a molecular sponge for miR-187 to up-regulate the bone morphogenetic protein 2 receptor antibody (*BMPR2*) [26]. In porcine follicles, *circINHA* as the ceRNA of miR-10a-5p protects granulosa cells against apoptosis by upregulating the connective tissue growth factor *CTGF* [27]. Chi-circ_ 0008219 regulates ovine follicular development by combining miR-468-3p, miR-84c-5p, and miR-483a [28]. Likewise, there are numerous reports regarding the circRNAs mediated regulation of gene expression in several biological processes [29,30]. Moreover, circRNAs expression patterns could be tissue-specific [31] to transcriptionally regulate the parental genes [29]. Also, recent studies have demonstrated that circRNAs are translated in a cap-independent manner [32].

Hanper sheep is a new breed of meat sheep raised by Hebei Animal Husbandry and Veterinary Research Institute and Hebei Agricultural University experts, the population has been cross and self-group breeding for 15 years and purebred to 6–8 generations, and its genetic performance is stable. Therefore, in order to improve the economic benefits of breeding, it is particularly important to improve their fertility. However, the mean lamb rate is 168%, which is higher than the 136% of Dorper sheep, but lower than the 260% of small-tailed Han sheep. The proportions of sheep that have 1, 2, or 3 or more lambs are 48.5%, 37.88%, and 13.62%, respectively. To explore effective breeding measures which could improve the regularity of fetal litter size in Hanper sheep, it is important to perform the differential expression analysis of ovarian circRNAs in the high- and low-fertility ewes. In this study, we collected follicles in the follicular phase and the corpus luteum tissues in the luteal phase from the ovaries of continuous monotocous (low reproduction) and polytocous (high reproduction) Hanper ewes respectively. Then, high-throughput sequencing was performed to screen the differentially expressed circRNAs (DE-circRNAs) and analysis functional enrichment of related genes. We aimed to reveal the candidate circRNAs and their target miRNAs that affect the fertility traits of Hanper sheep. Temporal and spatial expression models of circRNAs were constructed for better understanding and use of multi-lamb traits in sheep.

## 2. Materials and Methods

### 2.1. Ethics Approval

This study was authorized by the Animal Welfare and Ethical Committee of the Institute of Animal Sciences at the Hebei Agricultural University (IAS-CAAS, Baoding, China). All methods and procedures were performed on the experimental animals in accordance with the relevant guidelines (document approval number 2020070).

### 2.2. Selection of Experimental Animals and Sample Collection

Based on the lambing records of 1851 Hanper sheep (Hebei Liansheng Agricultural Development Co., Ltd. Zhuzhou, China), 12 unpregnant ewes aged 3–4 years were identified and divided into the monotocous group and the polytocous group, each of which consisted of six ewes. The ewes in the monotocous group lambed only one kid per litter within more than three consecutive litters. The ewes in the polytocous group lambed no less than three kids per litter within more than three consecutive litters. The natural estrus cycle of the 12 ewes was identified and the ewes were observed to have two normal natural estrus cycles in succession, then the bilateral ovaries of the ewes in the monotocous group (M) and the polytocous group (P) in the follicular phase (F) were collected at 36 h after the third natural estrus, denoted as MF and PF, respectively (three ewes in each group, six ewes in total). At 216 h (ninth day) after the third natural estrus, the bilateral ovaries of the ewes in the monotocous group (M) and the polytocous group (P) in the luteal phase (L) were collected, denoted as ML and PL, respectively (three ewes in each group, six ewes in total) (Table 1). The mesangium around the ovary was removed by disinfected scissors. These follicles whose diameter was greater than 3 mm in the follicular phase ovaries from the same individual were separated and placed separately in 2 mL RNase-free cryopreserved tubes. The luteal samples in the luteal phase ovaries from the same individual were separated and put separately into 2 mL RNase-free cryopreserved tubes. The tubes with the samples were immediately frozen in liquid nitrogen before transferring to the laboratory. After that, the samples were moved and stored at −80 °C for later use.

### 2.3. RNA Extraction and Library Construction

The follicles or luteum of 12 individual sheep tissues were frozen to grind into powder separately. Then, the total RNA in every sample was extracted with Trizol reagent (Thermo Fisher Scientific, Waltham, MA, USA). The concentration and purity of RNA were estimated by the RNA Nano 6000 Assay Kit with BioAnalyzer 2100 system (Agilent Technologies, Santa Clara, CA, USA) and 1% agarose gel electrophoresis, respectively.

A total amount of 5 μg RNA per sample was used as input material for the RNA sample preparations. Firstly, ribosomal RNA was removed by Epicentre Ribozero™ rRNA Removal Kit (Epicentre, Madison, WI, USA), and rRNA free residue was cleaned up by ethanol precipitation. Subsequently, linear RNA was digested with 3 U of RNase R (Epicentre, Madison, WI, USA) per µg of RNA. Sequencing libraries were generated by NEBNext^®^ Ultra™ Directional RNA Library Prep Kit for Illumina^®^ (NEB, Ipswich, MA, USA) following the manufacturer’s recommendations. Briefly, fragmentation was carried out using divalent cations under elevated temperature in the NEBNext First Strand Synthesis Reaction Buffer (5X). First strand cDNA was synthesized using random hexamer primer and M-MuLV Reverse Transcriptase (RNaseH-). Second strand cDNA synthesis was subsequently performed using DNA Polymerase I and RNase H. In the reaction buffer, dNTPs with dTTP were replaced by dUTP. The remaining overhangs were converted into blunt ends via exonuclease/polymerase activities. After adenylation of 3′ ends of DNA fragments, the NEBNext Adaptor with hairpin loop structure was ligated to prepare for hybridization. In order to select cDNA fragments of preferentially 150~200 bp length, the library fragments were purified with the AMPure XP system (Beckman Coulter, Beverly, IL, USA). Then 3 μL USER Enzyme (NEB, Ipswich, MA, USA) was used with size-selected, adaptor-ligated cDNA at 37 °C for 15 min followed by 5 min at 95 °C before PCR. Then PCR was performed with Phusion High-Fidelity DNA polymerase, Universal PCR primers, and Index Primer. At last, products were purified (AMPure XP system) and library quality was assessed using the Agilent Bioanalyzer 2100 system.

### 2.4. Sequence Mapping and CircRNA Prediction

The pure reads were mapped to the reference genome (Oar_v3.1) using the HiSAT2 alignment method which is a valid and quick tool to identify circRNA [33]. To ascertain the reliability of circRNAs, the BWA-MEM algorithm was applied for sequence segmentation and comparison, and the SAM file was used to find PCC (paired cut fragments), PEM (paired-end maps) sites, and GT-AG splicing signal [34]. Finally, the sequences with connection sites were reanalyzed using the dynamic programming algorithm, and circRNAs were annotated against the circBase database. CircRNAs without annotation were defined as novel circRNAs. Also, the type of exon chromosome and length distribution of the identified circRNAs were statistically analyzed.

### 2.5. Differential Expression Analysis of CircRNAs

Spliced Reads per Billion Mapping (SRPBM, defined as the number of circular reads/number of mapped reads read × length) was used to estimate the expression of circRNAs [35]. The DE-circRNAs were authenticated by the DEseq2 software package [36]. Since the chosen populations were identical sheep breeds and belonged to the same farm (Hebei Lian sheng Agricultural Development Co., Ltd. Baoding, Hebei, China), their growth characteristics were the same, only the number of litters was different. circRNAs with a fold change ≥ 1.5 and *p*-value < 0.05 between the two groups (MF vs. PF, ML vs. PL) were identified as the DE-circRNAs.

### 2.6. STEM Cluster and Weighted Gene Co-Expression Network Analysis (WGCNA) Analysis of CircRNAs

For clustering, comparison, and visualization, Short Time-series Expression Miner (STEM) software was used to display the trends in these two stages. All genes were divided into eight modules according to the expression pattern, and the *p*-value of each module was calculated using the Permutation Test method. The identified circRNAs with *p*-value < 0.05 were considered enriched. Furthermore, WGCNA analysis was performed [37]. According to the formula Amn = [(1 + Smn)/2] β, the similarity matrix was converted into an adjacency matrix, and then the adjacency matrix was converted into a topological overlap matrix (TOM). Meanwhile, the function diss Tom = 1-TOM was employed to invert the topological overlap matrix to obtain the dissimilarity matrix. The purpose of this exercise was to eliminate errors from background noise and pseudo-correlation. Finally, the function “hclust” was used to perform hierarchical clustering of the dissimilar matrix, and the Dynamic Tree Cut method was used to generate a cluster tree cut. This process merged genes with similar expression patterns on the same branch, and each branch represented a co-expression module, while different colors represented different modules. Correlation analysis and clustering were performed according to the FPKM values of the distinct genes. Next, we determined the target gene module through feature vector analysis and performed cluster analysis on the representative genes in all modules-module eigengene (ME). A higher correlation between ME indicates the higher relevance of the module.

### 2.7. Functional Analysis of CircRNAs in Follicular and Luteal Period

The host genes enrichment annotation of DE-circRNA was determined by the David and Kyoto Encyclopedia of Genes and Genomes (KEGG) database. Using the GOseq R software package, ClueGO, and Gene Set Enrichment Analysis (GSEA), Gene Ontology (GO) enrichment analysis was performed on the DE-circRNAs of host genes [38]. *p*-value < 0.05 denoted the significance of GO and KEGG pathways and their functional enrichment.

### 2.8. Analysis of MiRNA Target Sites

To further inquire into the functional roles of miRNA, putative targets were identified by the combination of three software, Mireap, Miranda (v. 3.3a), and TargetScan (v. 7.0) [39]. Cytoscape (v. 3.5.1) used the circRNA-miRNA negative interactions to construct the circRNA-miRNA networks.

### 2.9. Validation of CircRNA Expression

Nine DE-circRNAs (FDR ≥ 1.5, *p*-value < 0.05) and three miRNAs were stochastically selected for the follow-up verification. The details of circRNA-specific reverse splicing and miRNA primer sequences are shown in Table 2. Quantitative real-time polymerase chain reaction (qRT-PCR) was performed in the Roche lightcycler 480ii system (Roche Applied Science, Mannheim, Germany) using SYBR green real-time PCR master mix (TaKaRa, Dalian, China) following the manufacturer’s instructions. The reaction was executed at 95 °C for 3 min, followed by 40 cycles for 10 s at 95 °C and 30 s at 60 °C, respectively. GADPH and U6 were used as internal standards for circRNA and miRNA housekeeping genes, respectively. The relative change of gene expression among the control and experimental groups was calculated by the 2^−∆∆Ct^ method [40]. Also, the cyclization of circRNA was verified by Sanger sequencing [41].

## 3. Results

### 3.1. Genomic Characteristics of CircRNA in Sheep Ovaries

Based on agarose gel electrophoresis and UV spectrophotometry, the total amount of RNA and RIN value of all samples were optimal for circRNA database construction. In total, 12 libraries of MF (MF1, MF2, MF3), PF (PF1, PF2, PF3), ML (ML1, ML2, ML3), and PL (PL1, PL2, PL3) were sequenced using the Illumina novaseq 6000 platform. A total of 295,435,964 and 286,474,630 clean reads from MF and PF groups, and 260,102,794 and 295,142,936 clean reads from ML and PL groups were obtained respectively. A total of 4256 candidate circRNAs and 2184 host genes were identified with at least one “head-to-tail” splicing event (Table S1). Table 3 lists the 17 highly expressed circRNAs and their host genes related to sheep-follicle development in the four groups, including *SNX13*, *INSR*, *ARHGAP10*, *SLTM*, *ZEB1*, *UBE3A*, *USP3*, *ANKRD46*, *PDS5B*, *SLC30A7*, *CEP70*, *HERC4*, *KDM4C*, *ELF2*, *CEP120*, *REV3L*, and *RAP1B*. According to the splice site information of the circular RNA and the relative position of the gene structure. These circRNAs were divided into three types (Supplementary Figure S1A), exons (4057, accounting for 95.28%), introns (191, accounting for 4.48%), and intergenic (9, accounting for 0.21%).

We used Transcripts Per Million (TPM) map reads to normalize circRNA abundance. Among the normalized circRNAs, the transcript expression levels of each group (MF, PF, ML, PL) were kept similar. As shown in Supplementary Figure S1B, the TPM peak densities of MF, PF, ML, and PL ranged from 1–3 per million counts. Among the circRNAs in each group (MF, PF, ML, PL), the number of exons mainly ranged from 2 to 4, with a total of 3406 (Supplementary Figure S1C). Among them, the length of circRNA was mainly 300–400 nt (1415 in total) (Supplementary Figure S1D).

### 3.2. Diversity of CircRNA Isoforms and Circularization Forms

Since sheep data are not yet available in circBase databases, the identified circRNA can be considered new (Table S1). The 4256 identified circRNA were derived from 2184 loci, and most genes (1872, 80.11%) produced one to two circRNAs, 19.89% (465) produced 3–15 different circRNAs (Supplementary Figure S2A). For instance, the potential transforming growth factor binding protein 1 (*LTBP1*) can produce 15 different circRNAs (Supplementary Table S1). Notably, the circRNA spliced at the identical site contains a splicing signal, which usually consists of a GT dinucleotide (splice donor) at the 5′ end of the main spliceosome and an AG dinucleotide (splice acceptor) at the 3′ end. Given an AG acceptor splice site, there are usually multiple GT donor sites. In this study, transforming growth factor-beta receptor 3 (*TGFBR3*) produced three subtypes of circRNAs (novel_circ_0001803, novel_circ_0001805, and novel_circ_0001806; Figure S2B) with 3326, 8883, and 14,600 bp, respectively. Similarly, many circRNAs have different acceptors but share the same donor. In this study, the host gene *AKT* serine/threonine kinase 3 (*AKT3*) produced three subtypes of circRNAs that have three different acceptors but share a single donor NC_019469.2_31610710 (Supplementary Figure S2C). This showed that there were alternating and cross splicing events, where the parallel splicing site may join in multiple forward and reverse splicing reactions, or in coterminous exons distally placed exons.

In order to uncover the expression profiles of different circRNAs derived from the same host gene, we explored the expression abundance of circRNA subtypes at different developmental stages. Among the three subtypes of *TGFBR3* circRNA, novel_circ_0001805 was highly expressed in each group, but novel_circ_0001803 and novel_circ_0001806 were missed in the PF group (Supplementary Figure S2D). Likewise, for *AKT3* circRNA subtypes, novel_circ_0011336 was highly expressed in the MF and PL groups, while novel_circ_0011344 was highly expressed in the MF and PF groups (Supplementary Figure S2E). In brief, these results implied that reverse splicing promoted multiformity and functional complexity in circRNAs by generating the subtypes.

### 3.3. Identification of DE-CircRNAs

**Figure 1:**
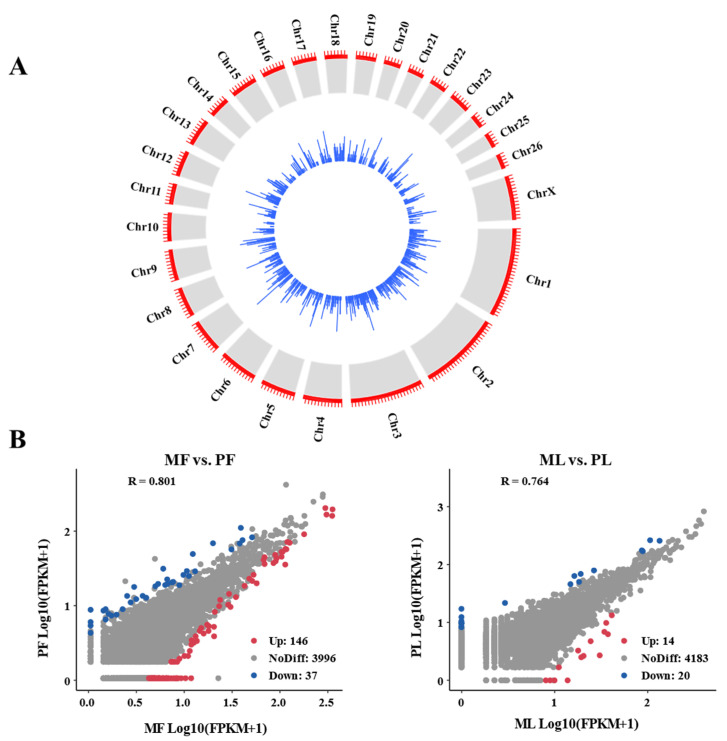
Differential expression of circRNAs. (**A**) The diagram showing the circRNA distribution and expression in chromosomes. The crosswise length of red lines and blue bar represents the number of circRNA transcripts in the set (The longer the transverse length, the more transcripts). The red ruler has the same length units. (**B**) Scatter plots of differentially expressed circRNAs. Red, blue, and gray dots in the graph represent transcripts that were significantly up-regulated, down-regulated, or unchanged respectively, which in monotocous sheep in the luteal phase versus polytocous sheep in the luteal phase (MF vs. PF), and in monotocous sheep in the follicular phase versus polytocous sheep in the luteal phase (ML vs. PL).

The distribution and expression of circRNAs on each chromosome are shown by a chord diagram (Figure 1A). In general, circRNAs are mainly distributed in chromosomes 1, 2, and 3. Ovarian DE-circRNAs in the polytocous and monotocous sheep were identified by DEseq2, based on criteria FDR ≥ 1.5 and *p*-value < 0.05. We found that there were 146 up- and 37 down-regulated circRNAs in MF vs. PF groups. Likewise, there were 14 up- and 20 down-regulated circRNAs in ML vs. PL groups, (Figure 1B) (Supplementary Table S2).

### 3.4. Functional Analysis of CircRNAs in Sheep of Varying Fecundity in the Follicular and Luteal Phases

**Figure 2:**
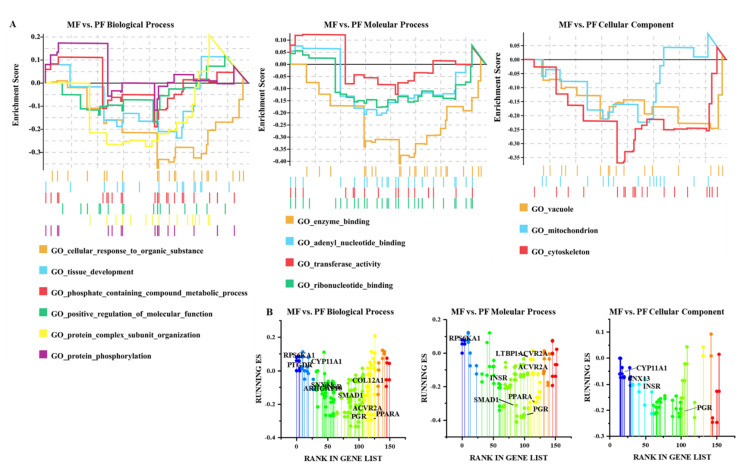
GSEA enrichment plots. (**A**) GSEA enrichment plots of GO terms which and in monotocous sheep in the luteal phase versus polytocous sheep in the luteal phase (MF vs. PF). (**B**) Rank-based gene set enrichment analysis of significant genes. Different colors represent different GO enrichment terms.

Based on the assumption that the functions of circRNA and host genes are known, we analyzed the pathways enrichment of the host genes producing DE-circRNAs to predict their potential functions during follicular development using GESA, GO, and KEGG. As shown in Figure 2A and Supplementary Figure S3, DE-circRNAs in MF vs. PF were mainly involved in the cellular response to endogen stimulus, phosphate-containing compound metabolic process, positive regulation of molecular function, protein complex subunit organization, tissue development, and metabolic process. The key cellular components were mitochondrion and vacuum, which performed molecular functions such as adenyl nucleotide binding, enzyme binding, transferase activity transferring phosphorus-containing groups, and carbohydrate derivative. The above enriched biological processes were mainly related to *PTGDR*, *CYP11A1*, *INSR*, *SMAD1*, *ACVR2A*, *RPS6KA001*, *COL12A1*, *PGR,* and *LTBP1* involved in regulating the follicular development of Hanper sheep (Figure 2B). As there were only 33 DE-circRNAs in ML vs. PL, GESA analysis of host genes could not be performed. Go Seq R enrichment analysis demonstrated that DE-circRNAs were mainly related to the biological processes include single-organism developmental process, acting on amino acids and derivatives, and cell surface composition (Supplementary Figure S4). *LTBP1*, *ITGAV*, *SNX13*, *CAAP1,* and *COL14A1* are they key DE-circRNAs that are involved in the regulation of follicle development (Supplementary Table S3).

**Figure 3:**
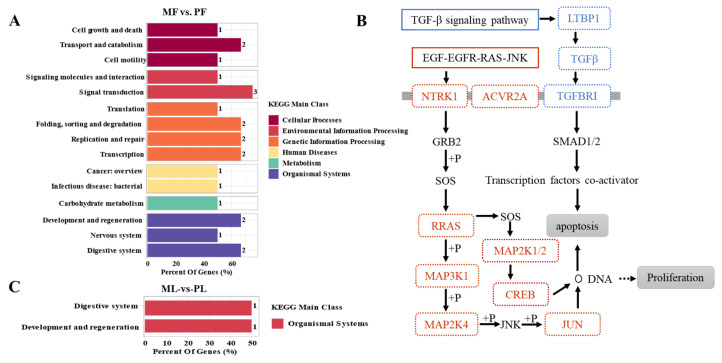
The top 20 KEGG enrichment analyses of differentially expressed circRNAs (DECs). (**A**,**B**) KEGG enrichment analysis which in monotocous sheep in the luteal phase versus polytocous sheep in the luteal phase (MF vs. PF). (**C**) KEGG enrichment analysis which in monotocous sheep in the follicular phase versus polytocous sheep in the luteal phase (ML vs. PL). Different colors represent different KEGG enrichment terms.

KEGG enrichment analysis of host genes of DE-circRNAs in MF vs. PF revealed the correlation between “Environmental Information Processing” and biological processes of “Organizational MAL Systems” (Figure 3A). Among these, the TGF-β signaling pathway, Rap1 signaling pathway, and cAMP signaling pathway involved in Environmental Information Processing were significantly enriched. Ovarian steroidogenesis, progesterone mediated oocyte maturation in Organizational MAL Systems, and the neurotrophin signaling pathway (Supplementary Table S4) were also significantly enriched. Finally, further analysis of the host genes involved in the enrichment pathway closely related to reproduction revealed that EGF-EGFR-RAS-JNK and TGF-β signaling pathways had important regulatory roles for sheep with different fertilities at the follicular stage (Figure 3B). KEGG enrichment analysis of DE-circRNAs host genes in ML vs. PL revealed that these were mainly enriched in the Organic Systems process (Figure 3C). As shown in Table 4, *ITGAV* participated in thyroid hormone and PI3K-Akt signaling pathway regulating the fecundity of Hanper sheep in ML vs. PL. Also, *COL14A1* mainly controlled the fecundity levels in Hanper sheep through protein digestion and absorption. In short, the enrichment processes in MF vs. PF and ML vs. PL are mainly related to the reproduction regulation genes involving *COL14A1*, *ITGAV*, *CYP11A1*, *ACVR2A,* and *SMAD1*.

### 3.5. STEM Cluster Analysis of DE-CircRNAs

**Figure 4:**
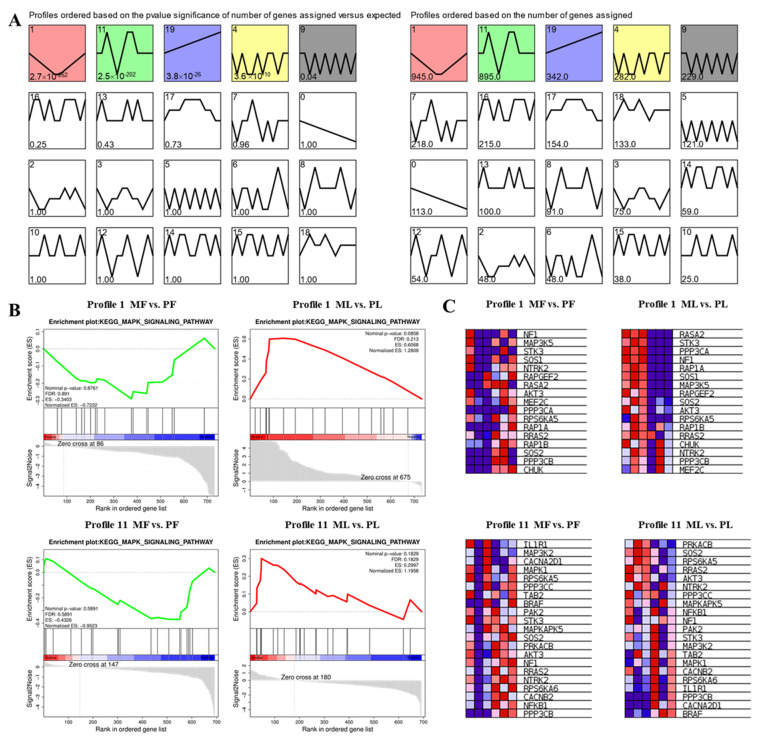
(**A**) Trend profiles produced by STEM across two phases. The profiles are placed according to the number of genes assigned. Colored profiles denote that gene expression was significantly enriched in those patterns, with a *Q* value of ≤0.05. In every pattern, the number of genes is revealed in the upper corner of the profile. (**B**) GSEA enrichment plots of KEGG signaling pathways. (**C**) Hierarchical clustering analysis of differential expressed host gene of circRNAs in Profile 1 and 11. Red and blue indicate the high and low expressions, respectively.

STEM software was used to perform trend analysis and 20 distribution chromatograms were constructed (Figure 4A). Profile 1 and Profile 11 which contain 945 and 895 cirRNAs host genes are two prominent profiles (Figure 4A). ClueGO results showed that host genes in profile 1 are mainly related to organelle organization, cellular macromolecule metabolic process, and anatomical structure morphogenesis biological processes (Supplementary Figure S5A, Supplementary Table S5). The genes of Profile 11 are mainly enriched in enzyme binding, and cellular macromolecule metabolic process (Figure S5B, Table S6). In addition, KEGG enrichment analysis using GESA showed that profile 1 and profile 11 are mainly enriched in the MAPK signaling pathway (Figure 4B). Moreover, Profile 1 mainly regulated follicular development in MF vs. PF and ML vs. PL through *MAP3K5*, *SOS1*, *AKT3*, and *SOS2* in the MAPK signaling pathway. Profile 11 mainly functions through the MAPK signaling pathway involving *IL1RL1*, *SOS2*, *AKT3*, *BRAF*, *MAPK1,* and *PAK2* to regulate follicle development in MF vs. PF and ML vs. PL (Figure 4C). Figure S6 revealed that the MAPK signaling pathways had important regulatory effects on sheep with different fertility and different stages in different circRNAs host genes of profile 1 and profile 11.

### 3.6. Construction of WGCNA Network

**Figure 5:**
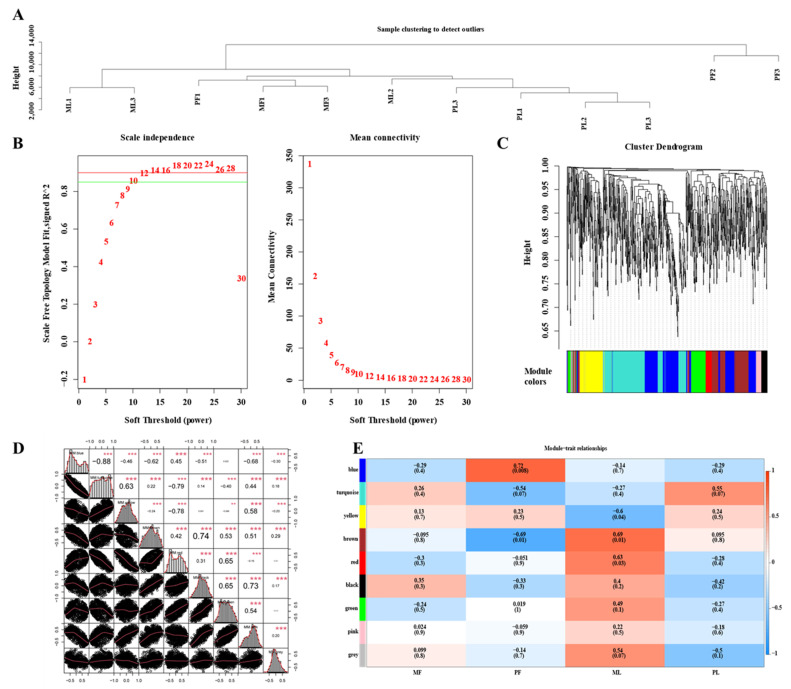
(**A**) Dendrogram of all 12 samples; (**B**) Soft threshold determination of gene co-expression network. The left panel shows the scale-free fit index (*y*-axis) as a function of the soft-thresholding power (*x*-axis). The right panel displays the mean connectivity (degree, *y*-axis) as a function of the soft-thresholding power (*x*-axis). (**C**) Shows a hierarchical cluster tree of coexpression modules identified by WGCNA. Every leaf on a tree is a gene. The main branches are made up of 9 modules, which are color-coded. (**D**) ME correlation between different modules. Black dots represent circRNAs, and the higher the value, the higher the correlation. The more and larger the asterisk, the greater the pairwise correlation between the modules. (**E**) Association analysis of gene co-expression network modules with tissues. Each row corresponds to a module, whose name is displayed on the left, and each column corresponds to a particular sample. The color of each cell at the row-column intersection indicates the correlation coefficient between the module and the sample. High correlations between specific modules and samples are shown in red.

In this study, 2212 high-expression genes were obtained by filtering out the low-expression genes. The results shown in Figure 5A indicate that there was no outlier in the gene cluster tree among all samples and therefore no further processing was required. R^2^ > 0.85, β = 14 was used as the soft threshold to construct the co-expression module (Figure 5B). After determining the soft threshold β = 9, Highly correlated genes were allocated to the same module (Figure 5C), and a total of nine co-expression modules were obtained. The genes in a module were assigned to a cluster based on their expression levels. Also, the genes with higher clustering were assigned to a module. The turquoise module contained the largest number of host genes (271) while the pink module contained the smallest number of host genes (20). The grey module is a set of genes that were not assigned to other modules (Figure S7). Furthermore, ME correlation analysis between two modules was carried out (Figure 5D). It was found that the ME correlation between the Brown and Black modules was 0.74, while it was 0.73 between the Black and Green modules. The correlation between the eigenvectors of nine modules and the sample sources was calculated (Figure 5E). The black module had the highest correlation in the MF group (COR = 0.35, *p*-value = 0.3), while the blue module had the highest correlation in the PF group (COR = 0.72, *p*-value = 0.008). The brown module exhibited the highest correlation in the ML group (COR = 0.69, *p*-value = 0.01), and the Turquoise module had the highest correlation in PL group (COR = 0.55, *p*-value = 0.07). Besides, the functional enrichment analysis of host genes in each module was carried out by David 6.8 online software, with *p* ≤ 0.05 as the significance threshold. Go analysis showed that genes in the blue module are mainly involved in membranes (*CAST*, *VEGFC*), chromatin binding (*SMAD6*), and mitochondrial membrane (*CYP11A1*), and genes in the black module are involved in protein phosphorylation (*SMAD5*, *PRKRA,* and *PPP3CB*) (Supplementary Table S7). Likewise, KEGG analysis revealed that the brown module genes are mainly enriched in the WNT signaling pathway. Green module genes are enriched in focal adhesion. Yellow module genes are enriched in the mTOR signaling pathway and PI3K-Akt signaling pathway. Turquoise module genes are enriched in the estrogen signaling pathway and insulin resistance (Table 5). In summary, host genes *AKT3* and were found to play key regulatory roles in multiple modules. The circRNAs corresponding to the above host genes are shown in Table S1.

### 3.7. MiRNA Targets of DE-CircRNAs

**Figure 6:**
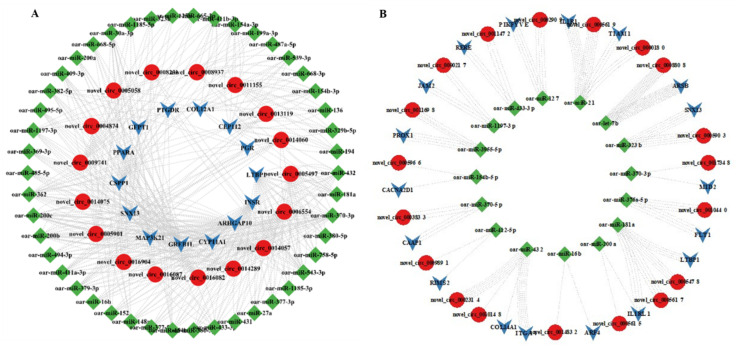
circRNA-miRNA interaction networks which in monotocous sheep in the luteal phase versus polytocous sheep in the luteal phase (MF vs. PF), and in polytocous sheep in the follicular phase versus monotocous sheep in the luteal phase (ML vs. PL). (**A**) MF vs. PF comparison, and (**B**) ML vs. PL comparison. Red, green, and arrows represent the circRNAs, miRNAs, and host genes, respectively.

CircRNA can indirectly regulate gene expression via the regulation of miRNA. In this study, we used miRanda software to predict miRNA binding sites in DE-circRNAs. In MF vs. PF, 18 of the 142 DE-CircRNAs have miRNA binding sites (Supplementary Table S8). In ML vs. PL, all of the 34 DE-CircRNAs have miRNA binding sites (Supplementary Table S9). As shown in Tables S7 and S8, most circRNAs had one or more miRNA binding sites. In MF vs. PF, novel_circ_0005497 (*LTBP1*) contained the largest number (10) of miRNA binding sites. In ML vs. PL, novel_circ_0016202 (*SPIRE1*) (spire type actin nucleation factor 1) contained up to 16 miRNA binding sites. Next, we used Cytoscape_v3.7 to construct a circRNA-miRNA negative regulatory network. As shown in Figure 6, 244 and 221 circRNA-miRNA pairs were identified in MF vs. PF and ML vs. PL, respectively. In MF vs. PF, the circRNA-miRNA interaction network involved 77 nodes (including 46 miRNAs and 17 circRNAs) and 418 edges (Figure 6A). It was found that oar-miR-27a, oar-miR-27a, oar-miR-16b, oar-miR-200a/b/c, oar-miR-181a, oar-miR-10a/b, and oar-miR-432 related to sheep fecundity were correlated with novel_circ_0005497 (*LTBP1*), novel_circ_0006554 (*INSR*), novel_circ_0016082 (*GREB1L*), novel_circ_0005617 (*IL1RL1*), novel_circ_0005884 (*SNX13*)*,* novel_circ_0014289 (*CYP11A1*), novel_circ_0008937 (*COL12A1*), novel_circ_0005617 (*IL1RL1*), novel_circ_0002309 (*ITGAV*), and novel_circ_0010140 (*COL14A1*). These results suggested that the DE-circRNAs play an important role in the regulation of Hanper sheep fertility. In ML vs. PL, the circRNA-miRNA interaction network involved 53 nodes (including 16 miRNAs and 19 circRNAs) and 138 margins (Figure 6B). It was found that oar-miR-370-5p, oar-miR-181a, oar-200a, oar-miR-323b, oar-miR-432, and oar-miR-16b were correspondingly correlated with novel_circ_0003833 (*CAAP1*), novel_circ_0005497 (*LTBP1*), novel_circ_0005617 (*IL1RL1*), novel_circ_0005884 (*SNX13*), novel_circ_0010140 (*COL14A1*), and novel_circ_0014832 (*ARF4*).

### 3.8. qRT-PCR Validation of DE-CircRNAs in the Regulatory Network

**Figure 7:**
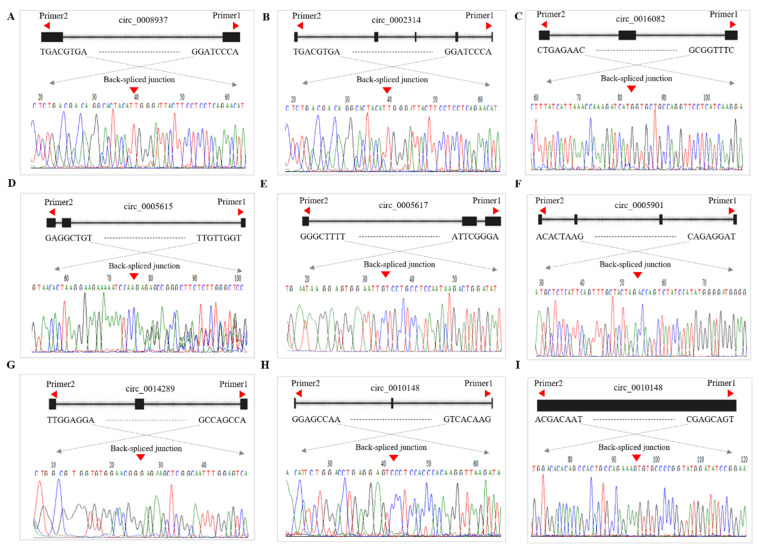
Schematic representation of circRNAs validation using divergent primer and sanger sequencing confirming head-to-tail splicing in circRNAs products. The black vertical line represents the back-spliced junction sites. Green and red horizontal lines indicate the 5′ and 3′ ends of the circRNA sequence, respectively. Note: (**A**–**I**) Divergent primers used in the amplification of circular junctions. Arrowheads in the left and right direction represent divergent primers. The downward red arrow represents the back spliced junction. The black squares represent exons. The curves in different colors represent the results of Sanger sequencing.

**Figure 8:**
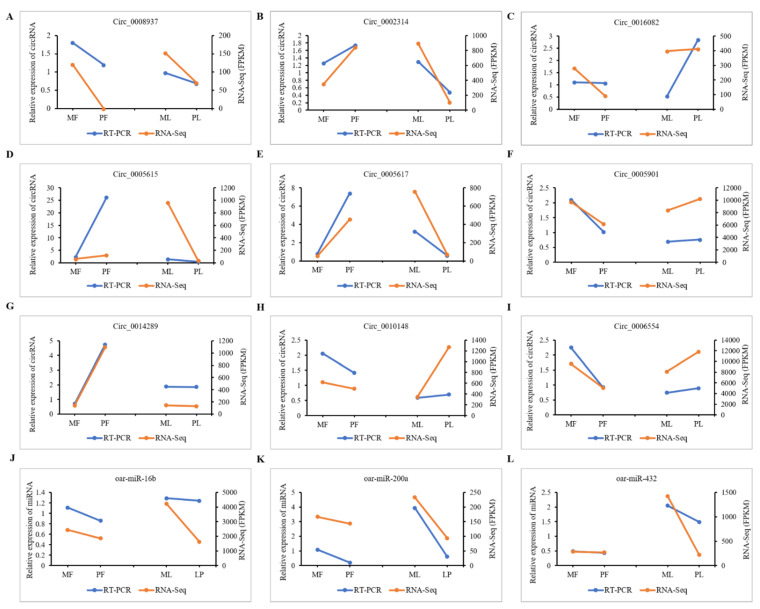
Validation circRNAs. (**A**–**I**) Experimental and sequencing validation of the 9 circRNAs. (**J**–**L**) Experimental and sequencing validation of the 9 miRNAs. Note: Blue and aurantium line represent RT-PCR and RNA-seq respectively.

Based on transcriptome sequencing, novel_circ_0008937, novel_circ_0002314, novel_circ_0005682, novel_circ_0005615, novel_circ_0005901, novel_circ_0014289, novel_circ_0010148, and novel_circ_0006554 are respectively, 1343, 8109, 2607, 3092, 2197, 6469, 4303, 13,549, and 552 bp (Supplementary Table S10). The corresponding sequence information is shown in Supplementary Table S8. The sequence information of the 9 qRT-PCR candidate circRNAs was retrieved from the NCBI database. The full-length sequences of novel_circ_0008937, novel_circ_0002314, novel_circ_0016082, novel_circ_0005615, novel_circ_0005617, novel_circ_0005901, novel_circ_0014289, novel_circ_0010148 and novel_circ_0006554 belongs to sheep *COL12A1*, *ITGAV*, *GREB1L, IL1RL1*, *IL1RL1*, *SNX13*, *CYP11A1*, *COL14A1*, and *INSR* genes which are circularized in exon base sequence of 45-46,14-18, 27-29, 5-7, 3-5, 1-4, 2-5, 2-4 and 2nd (Supplementary Figure S8), indicating that these circRNAs are mainly composed of exons. To determine whether the sheep circRNAs were looped correctly, a Divergent Primer was designed to amplify the linker sequences of 9 circRNAs (Supplementary Figure S8). 9 circRNA junction sequence products were obtained by PCR amplification using dispersed primers. Also, the presence of cyclization sites was affirmed by Sanger sequencing, which was consistent with Figure 7. The above results indicated that nine circRNAs were correctly ringed in the ovarian tissue of Hanper sheep. Then the nine cirRNAs novel_circ_0008937, novel_circ_0002314, novel_circ_0016082, novel_circ_0005615, novel_circ_0005617, novel_circ_0005901, novel_circ_0014289, novel_circ_0010148, and novel_circ_0006554 and the expression patterns of the three target miRNAs, oar-miR-16b, oar-miR-200a, and oar-miR-432 were verified by qRT-PCR in MF vs. PF and ML vs. PL. It was found that the expression pattern of these selected circRNAs during follicular development was highly consistent with the circRNA sequencing data (Figure 8).

## 4. Discussion

Fetal lambing traits are directly related to the follicle development of sheep ovaries [8]. Sheep fecundity, a complicated biological process, is tightly regulated by coordinated genes, covering coding and non-coding RNA. Notably, in the mammalian genome, 70–90% of non-coding RNAs are universally transcribed [42], indicating that they constitute the majority of the transcriptome. Transcriptome sequencing can uncover the genes related to reproductive traits and elucidate the underlying molecular mechanisms. In this study, we identified the DE-circRNAs in the ovaries of Hanper sheep and explored their target genes which may regulate ovarian development. We studied the circRNA genome characteristics in the follicles and corpus luteum of monotocous and polytocous Hanper sheep and found that most circRNAs had three or four exons, which was in agreement with previous findings in sheep skeletal muscle and uterus [8,17]. Besides, a total of 4257 circRNAs were identified between MF vs. PF and ML vs. PL. Interestingly, we found a total of 183 and 34 differentially expressed circRNAs in MF vs. PF. This shows a large difference in the expression patterns of circRNAs in ML vs. PL, indicating cell type-dependent characteristics of circRNA expression and differential regulation of circularization events in different cells. A similar phenomenon was also observed in other studies [42]. It is evident that cumulus cells are transcriptionally highly active in the process of follicular development. Follicular development could produce much more transcripts relative to the luteal tissues, and circRNA biogenesis mainly depends on the back-splicing of pre-mRNA transcribed in a cell [43]. We thus speculated that a larger amount of circRNAs produced in follicular tissues could be attributed to their higher transcriptional activity. About 20% of these circRNAs could produce more than one type of circRNA, which was lower than cows (40%) and honeybees (61%) [44]. This suggested that alternative splicing was indeed the main event in the formation of circRNAs [41]. We found that several known genes related to follicular development produced more than one circRNA. For instance, *TGFBR3* and *AKT3* had three predictive circRNAs, and *LTBP1* could produce up to 15 circRNAs. It seems that a parental gene can produce several circRNA subtypes, even more than 10 types in some cases. However, only one or two subtypes are prominent in general. Nevertheless, most other subtypes with low expression may play a specific function in the follicular development of Hanper sheep.

circRNAs are known to enhance the transcription of host genes by interacting with U1 small ribonucleoproteins (snRNPs) and RNA polymerase II [45]. Therefore, identifying the potential function of host genes associated with circRNA may reveal their function. In addition to the common host genes that affect reproductive performance such as *SMAD1*, *CYP11A1*, *INSR*, and *PGR*, we also identified potential members of the TGF-β binding protein family, which encoded for transfer growth factor binding protein 1 (*LTBP1*), located on sheep chromosome 3. Sheep *LTBP1,* with 29 exons and 28 introns, serves as the target of TGF-β and is usually released as an inactive complex from the cell. Studies show that TGF-β releases from epithelial cells requiring the proteolytic processing of *LTBP1*, thereby a cell cannot produce TGF-β in the absence of *LTBP1* [46]. This may affect cell maturation and differentiation [47]. Moreover, circRNAs also function as miRNA sponges and are involved in miRNA-mediated post-transcriptional regulation. However, only a limited number of circRNAs contain enough binding sites to bind specific miRNAs. For instance, circRNA ciRS-7 with more than 70 conserved binding sites acts as a sponge for miR-7 to regulate brain cell development [48]. On the contrary, recent evidence suggests that circRNA as a miRNA sponge does not require a large number of putative targeting sites. The circular RNA produced from exon 2 of the *HIPK3* gene has only two binding sites for miR-124 which is enough for inhibiting its activity [49]. Liang et al. show that *circSCIN* can be sponged with miR-133 and miR-148a/b, and its regulation determines the reproductive performance of Meishan and large white pigs [50]. *CirceGFR,* being a competitive endogenous RNA of miR-125a-3p, regulates *FYN* expression in mouse ovarian granulocyte cells [51]. Here, based on miRNA target site prediction analysis, we found that oar-miR-27a could be a target of novel_circ_0005497. miR-27a is known to inhibit the proliferation and promote apoptosis of granulosa cells in mice. This is achieved by reducing the key enzyme of estrogen synthesis, named aromatase [52]. In conclusion, *circLTBP1* is a potential competitive endogenous RNA (ceRNA) that may regulate gene transcription, and affect fertility traits as well as follicular development in sheep.

In addition to miRNA regulation, circRNA might have additional roles. Recent studies showed that circRNAs could also directly regulate protein synthesis through mRNA. Also, some of them can be converted into proteins for insertion via IRES (internal ribosome entry sites) [53]. Several factors and pathways, including TGFβ, PI3K-AKT, Wnt/β-catenin, and TGFβ-SMAD signaling pathways are related to follicular growth and development. Interestingly, Tao et al. showed that the host genes of ovarian follicles circRNAs participate in the generation of the ovarian corpus callosum and p53 signal transduction before ovulation in goats [54]. CircRNA host genes are also involved in the production of ovarian steroids and regulating their signals. These signals are essential for follicle growth, oocyte maturation, and ovulation. In this study, using STEM, WGCNA, and differential circRNA functional enrichment analysis, it was found that the signaling pathways of MAPK, Wnt, mTOR, PI3K-Akt, TGF-β, Rap1, and ovarian steroidogenesis were involved in circRNAs mediated regulation of sheep reproduction.

## 5. Conclusions

In this study, the circRNA expression profile of high and low litter size Hanper sheep was established. A total of 4752 circRNAs were identified, of which 183 circRNAs were differentially expressed in MF vs. PF and 34 circRNAs were differentially expressed in ML vs. PL. In total, 17 key circRNAs involved in the development of ovine follicles were identified, and they function through the signaling pathways of MAPK, WNT, mTOR, PI3K-AKT, TGF-β, RAP1, EGF-EGFR-RAS-JNK, and ovarian steroidogenesis to regulate the development of sheep follicles. Also, the target miRNA sites in circRNAs for oar-miR-27a, oar-miR-16b, oar-miR-200a/b/c, oar-miR-181a, oar-miR-10a/b, oar-miR-432 were revealed through competitive endogenous RNA network analysis. It seems that circRNAs mainly function as sponges of several reproductive-related miRNAs, which in turn affects the fetal litter traits of Hanper sheep.

## Figures and Tables

**Table 1:** The basic information on litter size.

Group	Without Pregnancy Sheep (Earmark)	Litter Size			
		First Parity	Second Parity	Third Parity	Four Parity
Polytocous group	3E202	3	3	5	
	6T01	3	4	2	
	6T06	3	3	4	3
	CE104	3	3	3	
	3E161	3	3	4	
	CE103	3	4	3	4
Monotocous group	9M06	1			
	MY91				
	MY86				
	3E146				
	3E79				
	MY66				

**Table 2:** Quantitative real-time PCR primer sequences.

Type	Gene Name	Primer Sequences (5′-3′)	Amplicon Size (bp)
CircRNA	novel_circ_0006554	F: ACGACAATGAGGAGTGTGGG	198
		R: GTCCTTCAATGACCGAGCAGT	
	novel_circ_0005901	F: ACACTAAGTGATGACGAATCTTTTC	190
		R: CCACCCACAAAGCAGAGGAT	
	novel_circ_0008937	F: TGACGTGAATGTCTATGCTCAGT	153
		R: GGAGAGGGTGAAGGATCCCA	
	novel_circ_0016082	F: CTGAGAACCAACAGCAGTGGA	187
		R: AGCTCTTCCAGGCGGTTTC	
	novel_circ_0002314	F: GCTGCTGATGCAACAGGGTT	198
		R: CAGGCAGAGGGCAGGTTTTA	
	novel_circ_0005615	F: GAGGCTGTAACGGAAGAGGA	199
		R: AGCACGTTAGGTTTGTTGGT	
	novel_circ_0005617	F: GGGCTTTTCTTTGCCTCCTG	199
		R: TTGTGGGACAAAATATTCGGGA	
	novel_circ_0010148	F: GGAGCCAAAACCCAGAGTCAA	187
		R: ACCTGAAGCTGGAGTCACAAG	
	novel_circ_0014289	F: TTGGAGGATGTCAAGGCCAA	180
		R: TACTGGTGATAGGCCAGCCA	
Control	GADPH	F: ACAGTCAAGGCAGAGAACGG	107
		R: CCAGCATCACCCCACTTGAT	
miRNA	oar-miR-16b	GCGCGTAGCAGCACGTAAA	
	oar-miR-200a	CGCGCGAACACTGTCTGGT	
	oar-miR-432	CGCGTCTTGGAGTAGGTCATT	
Control	U6	AGTGCAGGGTCCGAGGTATT	

**Table 3:** The top 17 expressed circRNAs in MF vs. PF and ML vs. PL.

ID	Host_Gene_Name	MF. TPM	PF. TPM	ML. TPM	PL. TPM
novel_circ_0005901	*SNX13*	527	151	377	663
novel_circ_0006554	*INSR*	533	122	343	774
novel_circ_0014057	*ARHGAP10*	457	129	330	615
novel_circ_0008317	*SLTM*	444	150	220	504
novel_circ_0012048	*ZEB1*	410	221	165	264
novel_circ_0014135	*UBE3A*	334	164	135	335
novel_circ_0008261	*USP3*	426	207	291	450
novel_circ_0009916	*ANKRD46*	273	94	212	335
novel_circ_0010401	*PDS5B*	271	69	199	292
novel_circ_0001969	*SLC30A7*	189	103	118	175
novel_circ_0001127	*CEP70*	254	82	288	397
novel_circ_0016729	*HERC4*	198	89	159	249
novel_circ_0003675	*KDM4C*	190	107	109	223
novel_circ_0013646	*ELF2*	221	78	158	264
novel_circ_0006681	*CEP120*	168	88	169	238
novel_circ_0009038	*REV3L*	133	29	70	156
novel_circ_0004301	*RAP1B*	191	50	109	233

The numbers in each column denote the expression level of circRNA in descending order. Red and green denote the higher and lower expression levels of circRNAs, respectively. Also, the lighter the color, the lower is the circRNA TPM value. Note: Normalized expression level in TPM = (read count × 1,000,000)/libsize (libsize is the sum of circRNA read count). MF: Monotocous sheep in the follicular phase, PF: Polytocous sheep in the follicular phase, ML: Monotocous sheep in the luteal phase, PL: Polytocous sheep in the luteal phase.

**Table 4:** KEGG enrichment analysis of all differentially expressed transcripts in ML vs. PL.

Pathway Name	*p*-Value	Genes
Cell adhesion molecules (CAMs)	0.01	*ITGAV*, *JAM2*
Glycosaminoglycan degradation	0.02	*ARSB*
Dorso-ventral axis formation	0.02	*SPIRE1*
Arrhythmogenic right ventricular cardiomyopathy (ARVC)	0.06	*ITGAV*
Hypertrophic cardiomyopathy (HCM)	0.06	*ITGAV*
Dilated cardiomyopathy	0.07	*ITGAV*
ECM-receptor interaction	0.07	*ITGAV*
Small cell lung cancer	0.08	*ITGAV*
Protein digestion and absorption	0.09	*COL14A1*
Thyroid hormone signaling pathway	0.10	*ITGAV*
Lysosome	0.10	*ARSB*
Leukocyte transendothelial migration	0.10	*JAM2*
Tight junction	0.11	*JAM2*
Phagosome	0.13	*ITGAV*
Focal adhesion	0.16	*ITGAV*
Proteoglycans in cancer	0.17	*ITGAV*
Regulation of actin cytoskeleton	0.17	*ITGAV*
Pathways in cancer	0.25	*ITGAV*
PI3K-Akt signaling pathway	0.26	*ITGAV*
Metabolic pathways	0.66	*ARSB*

**Table 5:** KEGG enrichment analysis of the target modules.

Module Color	KEGG Term	*p*-Value	Gene
Blue	Ubiquitin mediated proteolysis	3.50 × 10^−2^	*FBXW7*, *HUWE1*, *BIRC6*, *UBE2Q2*, *UBA6*
	Ras signaling pathway	4.40 × 10^−2^	*RAB5C*, *TIAM1*, *PDGFD*, *RRAS2*, *STK4*
Black	Leishmaniasis	9.40 × 10^−2^	*TAB2*, *TGFB2*
Brown	Wnt signaling pathway	5.90 × 10^−2^	*APC*, *LRP6*, *ROCK2*
Green	Focal adhesion	3.20 × 10^−2^	*AKT3*, *DOCK1*, *ITGB1*, *LAMC1*, *MAPK1*
	Toxoplasmosis	5.60 × 10^−2^	*AKT3*, *ITGB1*, *LAMC1*, *MAPK1*
	mTOR signaling pathway	1.30 × 10^−2^	*AKT3*, *RICTOR*, *MAPK1*
	PI3K-Akt signaling pathway	1.90 × 10^−2^	*AKT3*, *ITGB1*, *LAMC1*, *MAPK1*, *PPP2R2A*
	Estrogen signaling pathway	3.50 × 10^−2^	*AKT3*, *FKBP5*, *MAPK1*
	TNF signaling pathway	4.20 × 10^−2^	*AKT3*, *MAPK1*, *RIPK1*
Turquoise	Endocytosis	1.50 × 10^−2^	*WASL*, *LDLRAP1*, *NEDD4L*, *PSD3*, *RABEP1*, *STAM2*
	Insulin resistance	2.70 × 10^−2^	*GFPT1*, *PPARA*, *PIK3R1*, *PRKAA2*, *PRKCE*
	Neurotrophin signaling pathway	3.70 × 10^−2^	*ABL1*, *RAP1B*, *NTRK2*, *PIK3R1*, *RPS6KA5*
	Fc gamma R-mediated phagocytosis	5.60 × 10^−2^	*WASL*, *PIK3R1*, *PLPP1*, *PRKCE*
	Notch signaling pathway	9.90 × 10^−2^	*NUMBL*, *SNW1*, *RBPJ*
	T cell receptor signaling pathway	0.012	*FYN*, *SOS1*, *DLG1*, *TEC*
Yellow	Focal adhesion	0.018	*FYN*, *ARHGAP5*, *SOS1*, *IGF1R*
	PI3K-Akt signaling pathway	0.025	*RHEB*, *SOS1*, *GYS1*, *IGF1R*
	Adherens junction	0.044	*FYN*, *IGF1R*, *PTPRM*

## Data Availability

The data presented in this study are available on request from the corresponding author.

## Supplementary Materials

The following are available online at https://www.mdpi.com/article/10.3390/ani11071863/s1, Figure S1: circRNAs characteristics in sheep ovary. (A) Percentages of the three forms of circRNAs. (B) The expression levels of circRNAs based on TPM. (C) Number of exons in each transcript of sheep circRNA. (D) Exon length distribution of circRNAs; Figure S2. (A) cirRNAs distribution among host genes in sheep. (B) Three receptors and one donor position of *TGFBR3* in the genome. (C) Three donors and one receptor position of *AKT3* in the genome. (D–E) *TGFBR3* and *AKT3* expression in the four stages of sheep follicular development. Note: Normalized expression level in TPM= (read count × 1,000,000)/libsize (libsize is the sum of circRNA read count); Figure S3. The top 20 GO enrichments of differential expressed circRNAs (DE-circRNAs) in polytocous sheep in the luteal phase versus monotocous sheep in the luteal phase (MF vs. PF); Figure S4. The top 20 GO enrichments of differential expressed circRNAs (DE-circRNAs) in polytocous sheep in the follicular phase versus monotocous sheep in the luteal phase (ML vs. PL); Figure S5. The network plot showed the biological process GO terms produced by ClueGO in the profiles generated by STEM; Figure S6. MAPK signaling pathways at different stages in different circRNAs host genes of profile 1 and profile 11; Figure S7. Distribution of the number of genes in modules. Figure S8. Sequences information. Table S1. Identified circRNA; Table S2. Different expression circRNA; Table S3. CAD_GO_enrichment_result; Table S4. KEGG enrichment analysis of all differentially expressed transcripts in MF vs. PF; Table S5. ClueGO analysis in MF vs. PF group; Table S6. ClueGO analysis in ML vs. PL group; Table S7. GO enrichment result of the target modules; Table S8. MF vs. PF. miRNAbindingsite.binding_site; Table S9. ML vs. PL. miRNAbindingsite.binding_site; Table S10. Sequences information.
