# Supplementary material for: Differential Expression and Functional Analysis of CircRNA in the Ovaries of Low and High Fecundity Hanper Sheep
Source: Animals (Basel). 2021 Jun 23;11(7):1863. doi: 10.3390/ani11071863 (PMC8300399; doi:10.3390/ani11071863)
Supplement: Supplementary file 1 [file animals-11-01863-s001.zip › Supplement figure.pdf]

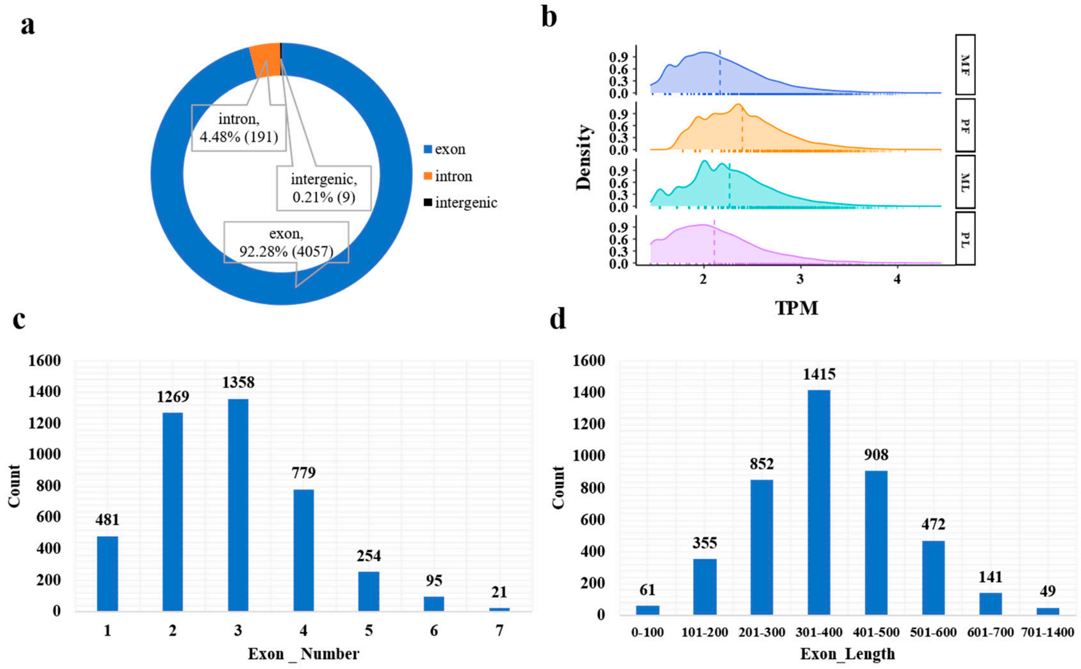

Supplementary Figure S1.

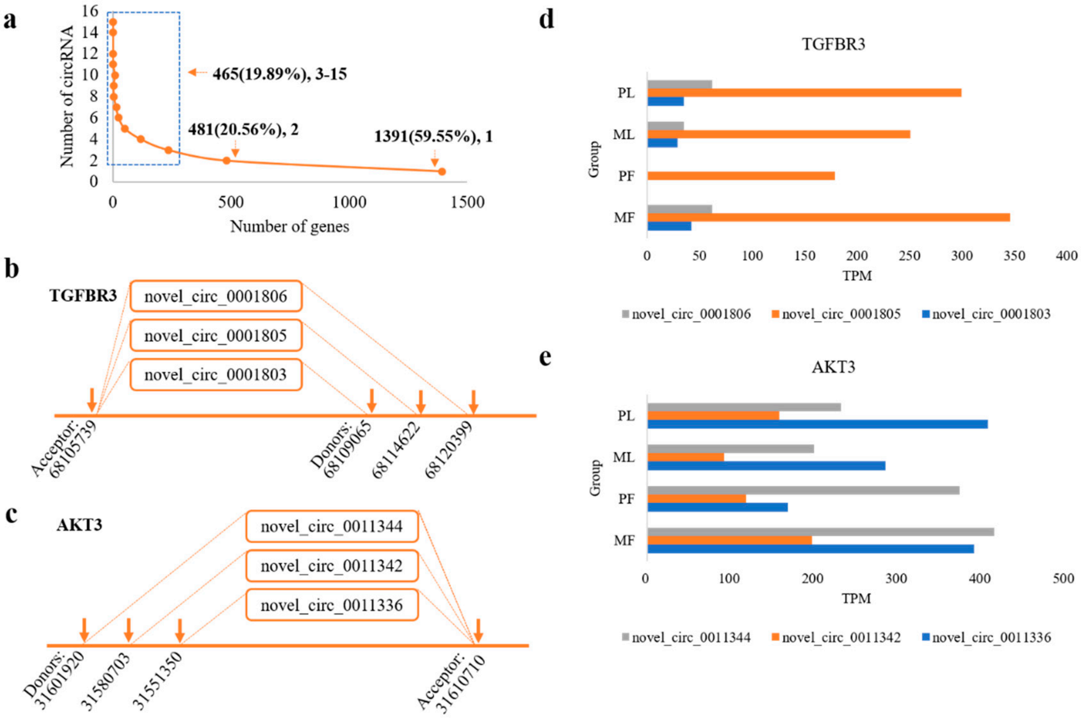

Supplementary Figure S2.

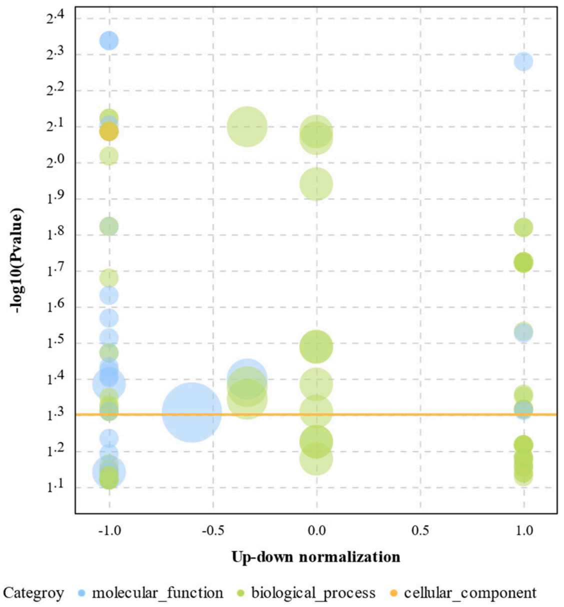

Top 20

ID	Descrption
GO:0016855	racemase and epimerase activity
GO:0036361	racemase activity
GO:0004764	shikimate 3-dehydrogenase (NADP+) activity
GO:0007517	muscle organ development
GO:0061061	muscle structure development
GO:0016717	oxidoreductase activity
GO:0048856	anatomical structure development
GO:0008156	negative regulation of DNA replication
GO:0048478	replication fork protection
GO:0051053	negative regulation of DNA metabolic process
GO:0090329	regulation of DNA-dependent DNA replication
GO:2000104	negative regulation of DNA-dependent DNA replication
GO:0048646	anatomical structure formation involved in morphogenesis
GO:0009986	cell surface
GO:0048731	system development
GO:0045005	DNA-dependent DNA replication maintenance of fidelity
GO:0048468	cell development
GO:0017089	glycolipid transporter activity
GO:0046836	glycolipid transport
GO:0045766	positive regulation of angiogenesis

Supplementary Figure S3.

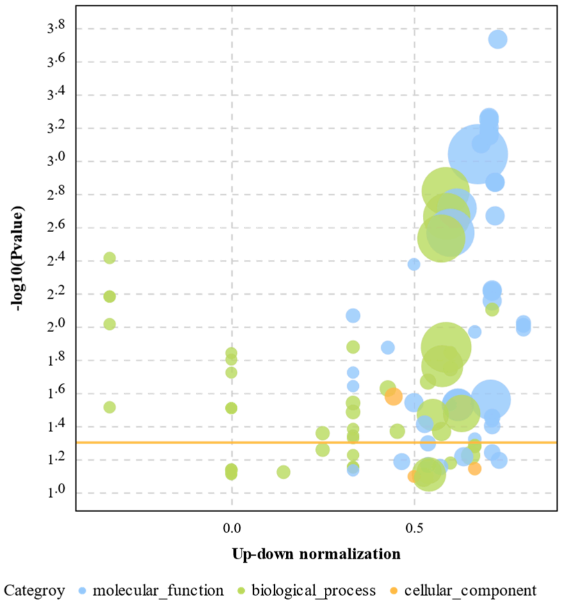

Top 20

ID	Descrption
GO:0097367	carbohydrate derivative binding
GO:0035639	purine ribonucleoside triphosphate binding
GO:0001883	purine nucleoside binding
GO:0032549	ribonucleoside binding
GO:0032550	purine ribonucleoside binding
GO:0032555	purine ribonucleotide binding
GO:0001882	nucleoside binding
GO:0017076	purine nucleotide binding
GO:0032553	ribonucleotide binding
GO:0043168	anion binding
GO:0005488	binding
GO:0000166	nucleotide binding
GO:1901265	nucleoside phosphate binding
GO:0008152	metabolic process
GO:0043167	ion binding
GO:0036094	small molecule binding
GO:0044238	primary metabolic process
GO:0003824	catalytic activity
GO:0071704	organic substance metabolic process
GO:0007606	sensory perception of chemical stimulus

Supplementary Figure S4.

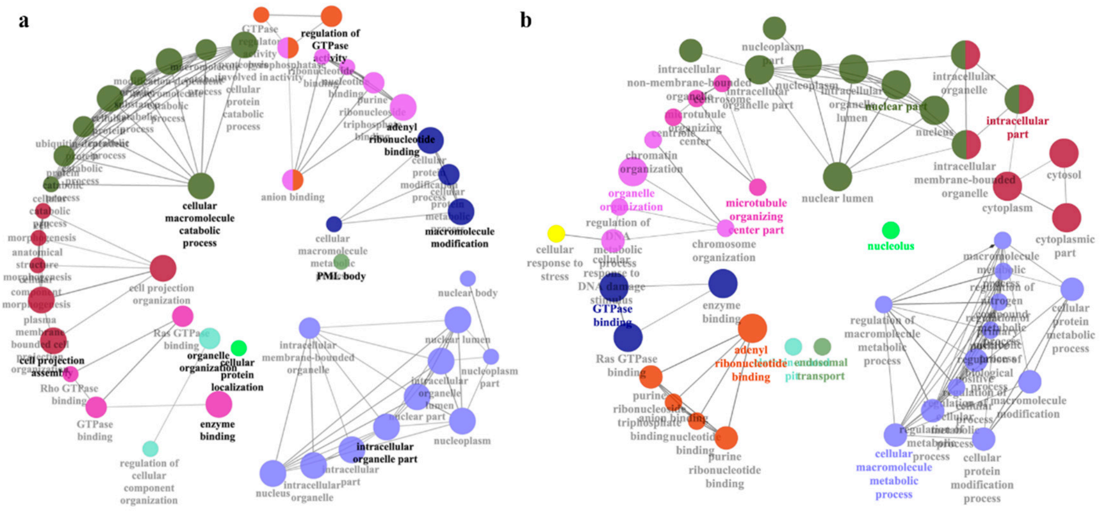

Supplementary Figure S5.

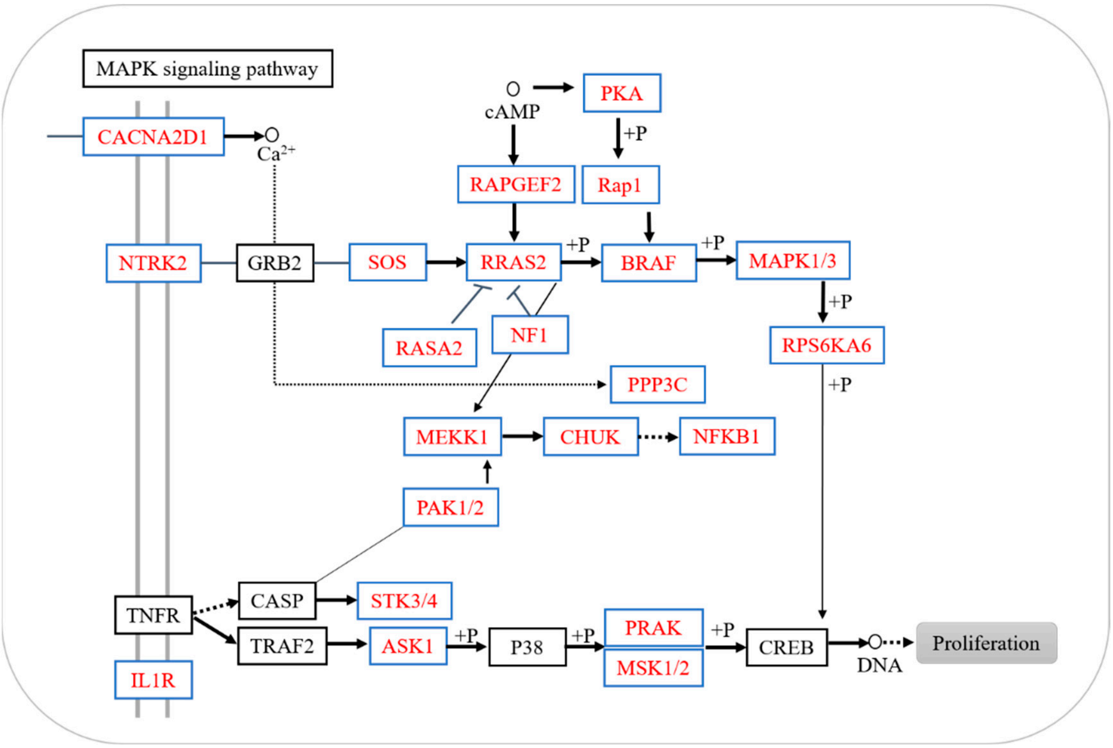

Supplementary Figure S6.

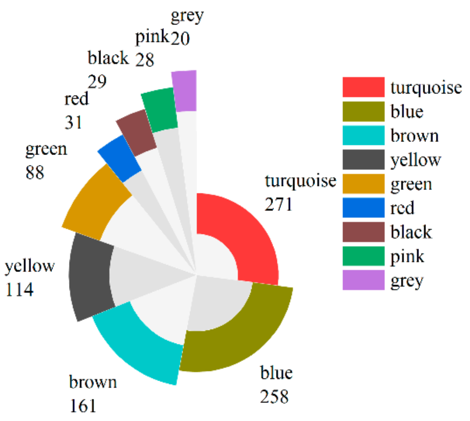

Supplementary Figure S7.

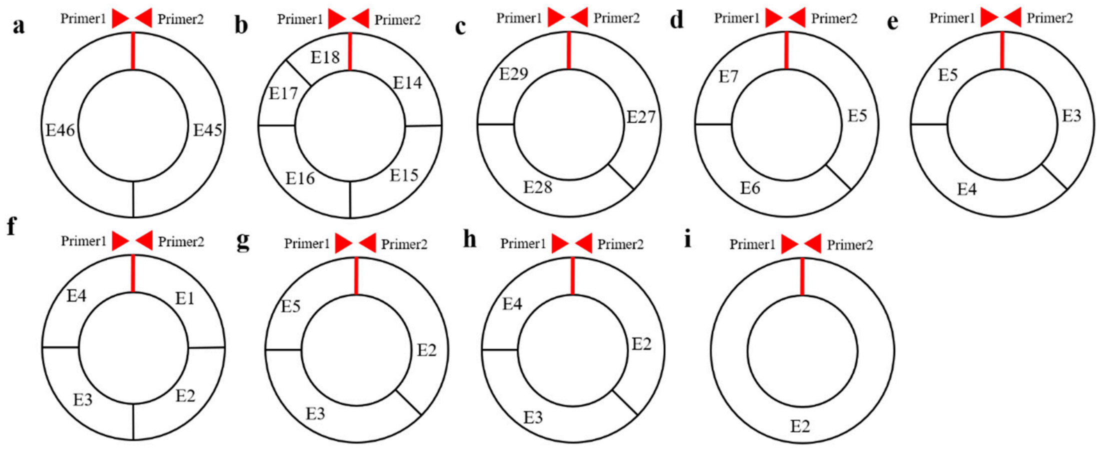

Supplementary Figure S8.
